# An increase in somatization in pandemic time in association with lexical characterictic of statements about pandemic

**DOI:** 10.1192/j.eurpsy.2022.1235

**Published:** 2022-09-01

**Authors:** T. Medvedeva, S. Enikolopov, O. Boyko, O. Vorontsova, M. Stankevich

**Affiliations:** 1 Federal State Budgetary Scientific institution “Mental health research center”, Clinical Psychology, Moscow, Russian Federation; 2 Federal Research Center ”Computer Science and Control” of RAS, Institute For Systems Analysis, Moscow, Russian Federation

**Keywords:** lexical analysis of statements, somatization, quarantine, Covid-19

## Abstract

**Introduction:**

One of the negative consequences of the COVID-19 pandemic may be an increase in somatization.

**Objectives:**

identification of implicit characteristics of texts indicating the peculiarities of the opinion about the pandemic by people with high somatization level.

**Methods:**

Survey (03/23/2020–01/29/2021, N=1188). Used: SCL-90-R, COPE, Constructive Thinking Inventory (CTI). It was offered to express an opinion on the pandemic. The statements were divided into the two text arrays - “high somatization” and “low somatization” (based on the parameter “somatization” SCL-90R). The frequency of words in these text arrays was estimated (LIWC).

**Results:**

The analysis showed an increase in somatization as the pandemic developed (Std.J-T Statistic=4,327). The relationship between somatization and anxiety, sleep disturbances, and depression was revealed. Higher rates of somatization are associated with a decrease in emotional coping, «global constructive thinking» and «personal superstitious thinking», an increase in «categorical thinking». The connection between somatization and a number of non-constructive copings is shown. Texts associated with high somatization demonstrate higher number of pronouns of the first person (30.77%, 17.19%), a decrease in the tonality of words, a vocabulary (LIWC) of suffering, negative sthenic emotions (1,53%, 0,93%), a decrease in the vocabulary of motivation and resistance (0,93%, 1,49%), a decrease in vocabulary associated with the body (0,20%, 0,32%).

**Conclusions:**

The connection between somatization and high emotional distress, which manifests itself in negative emotional vocabulary and is associated with a low level of emotional coping, is shown. The “representation” of the pandemic, presented in the text, is “divorced” from somatic manifestations, fear of illness and death.

**Disclosure:**

No significant relationships.

